# Crowdsourcing smartphone data for biomedical research: Ethical and legal questions

**DOI:** 10.1177/20552076231204428

**Published:** 2023-10-03

**Authors:** Michael Lang, Kyle McKibbin, Mahsa Shabani, Pascal Borry, Vincent Gautrais, Kamiel Verbeke, Ma’n H Zawati

**Affiliations:** 1 Faculty of Medicine and Health Sciences, 574124 Centre of Genomics and Policy, McGill University, Montreal, Canada; 2 Faculty of Law and Criminology, Ghent University, Institute for International Research on Criminal Policy, Ghent, Belgium; 3 26657 KU Leuven, Centre for Biomedical Ethics and Law, Leuven, Belgium; 4 33502 Université de Montréal, Faculté de droit, Chaire L.R. Wilson sur le droit des technologies de l’information et du commerce électronique, Montreal, Canada

**Keywords:** smartphone, biomedical research, medical data

## Abstract

The use of smartphones has greatly increased in the last decade and has revolutionized the way that health data are being collected and shared. Mobile applications leverage the ubiquity and technological sophistication of modern smartphones to record and process a variety of metrics relevant to human health, including behavioral measures, clinical data, and disease symptoms. Information processed by mobile applications may have significant utility for increasing biomedical knowledge, both through conventional research and emerging discovery paradigms such as citizen science. However, the ways in which smartphone-collected data may be used in nontraditional modes of biomedical discovery are not well understood, such as using data to train artificially intelligent algorithms and for product development purposes. This paper argues that the use of mobile health data for algorithm training and product development is (a) likely to become a prominent fixture in medicine, (b) likely to raise significant ethical and legal challenges, and (c) warrants immediate scrutiny by policymakers and scholars. We introduce the concept of “smartphone-crowdsourced medical data,” or SCMD, and set out a broad research agenda for addressing concerns associated with this new and potentially momentous practice. We conclude that SCMD for algorithm training raises a number of ethical and legal issues which require further scholarly attention to ensure that individual interests are protected and that emerging health information sources can be used in ways that maximally, and safely, promote medical innovation.

## Background

Smartphones have proliferated greatly in the last decade and have revolutionized how health data are being collected and shared.^1^ Mobile applications leverage the ubiquity and technological sophistication of modern smartphones to record and process a variety of metrics relevant to human health, including behavioral measures, clinical data, and disease symptoms.^2^ App-facilitated data collection is often enabled by wearables and smart devices, such as smart watches, smart speakers, and mobile sensors installed in the home or workplace.^3^ These devices, which are also increasing rapidly in popularity, are often powered by or connected to a smartphone application. Because nearly five billion people around the world own mobile devices,^4^ more than half of which have been used to collect and process health-related data,^5^ the existing volume of potentially exploitable health data is unprecedentedly large and growing rapidly. Apps may engage in data collection in various ways, including *actively*, through user surveys and manual data entry, or *passively*, by using built-in sensors and device software. Genetics data may have special salience in mobile health: direct-to-consumer genetics testing firms and third-party service providers are increasingly developing mobile applications to offer genetic testing services, interface with customers, and communicate variant interpretations.^6^

All of the information processed by these kinds of applications could have significant utility for increasing biomedical knowledge, both through conventional research and emerging discovery paradigms such as citizen science, in which amateur or nonprofessional scientists engage in research.^7^ And though much progress has been made in recent years toward addressing some of the legal and ethical implications of using smartphone data for formal, organized research,^8^ less is understood about the ways smartphone-collected data could be used in nontraditional modes of biomedical discovery. One possibility is that data processed by mobile health applications will be repurposed for training artificially intelligent algorithms in the medical context or elsewhere.^9^ Possible applications of this could include the development of algorithms that predict medical diagnoses^10^ or forecast psychological well-being.^11^ As the role of artificial intelligence (AI) in data-intensive fields continues to expand, there will inevitably be increasing demand for data inputs to train and refine algorithms that provide medical services, assist in the discovery of new treatments, and direct patient care. Mobile health data represent a unique and as yet largely unexplored opportunity to assemble datasets of enormous size and diversity that may serve as a productive training ground for AI.

However, it is difficult to know whether such data are already being used for these purposes. Health applications are often vague about how they plan to use, and share, collected data.^12^ Terms of service might alert app users to data processing for “quality control” or “product development,” which in some cases may function as subtle allusions to algorithm design and training. Even where planned uses are clearly demarcated, they are often communicated in lengthy or unapproachable consent materials, leading app users to consent to uses they do not fully appreciate. As both AI and mobile health come to occupy ever more significant roles in healthcare and biomedical research, it will be important to understand whether and in what manner these technological developments might operate in tandem. This paper argues that the use of mobile health data for algorithm training and product development is (a) likely to become a prominent fixture in medicine, (b) likely to raise significant ethical and legal challenges, and (c) warrants immediate scrutiny by policymakers and scholars. We introduce the concept of “smartphone-crowdsourced medical data,” or SCMD, and set out a broad research agenda for addressing concerns associated with this new and potentially momentous practice.

### 1. Smartphone-crowdsourced medical data (SCMD)

Data collected and processed by smartphone applications might, as we suggested above, have exceptional aggregate value. Though a single individual's app-processed health data likely has little scientific or commercial utility on its own, data collected across a population of hundreds, or thousands, of app users could contribute enormously to the advancement of biomedical knowledge. We refer to the aggregation of mobile health data for training algorithms that contribute to biomedical research or health product development as “smartphone-crowdsourced medical data” (SCMD). In practice, the aggregation of app user data might occur in one of several ways.

One possibility is that data collected by a single app will feed into the development of a single dataset comprised of the aggregate information of multiple users. An example of this can be seen in work conducted by Sophie Attwood and colleagues, which surveys the potential of a mobile app to reduce alcohol consumption. A single application, *Drinkaware*, was used in the context of this study to collect self-reported data from over 100,000 individuals over a 13-month period.^13^ Another model would be one in which data collected by multiple apps feeds into one dataset. An app developer, for example, could publish several apps or utilize wearables and sensors to process distinct kinds of medical data which could the be aggregated in a single dataset. A possible example of this can be seen in perspective work outlined by Lisa Marzano and colleagues, who describe how automated data collection from several sources could be used in the mental health research context to triangulate “a fine-grained and ecologically valid picture of an individual's emotional state and associated behaviour.”^14^

A third possibility is that multiple apps collecting medical data for multiple datasets could be independently accessed and aggregated in a separate setting for training or research purposes. This could happen if custodians of app-collected data make user information available to external entities through a formal data sharing regime or commercially oriented data brokerage. For our purposes, these distinctions can be treated as relatively minor variations on a core theme: that app-collected data are assembled *en masse* and could be used for the goal of increasing biomedical knowledge and biomedical research.

We imagine that SCMD will sometimes be collected for a primary function other than medical algorithm training. A fitness tracking app that collects personal health information for the interest and amusement of its users, for example, could simultaneously function to aggregate a diverse dataset amenable to repurposing. App users probably would not imagine that their innocuous personal fitness information could be useful for medical research or algorithm training—they may not even realize that they have consented to these kinds of uses. Importantly, we also imagine that a certain number of applications will engage in data collection explicitly and primarily for the kinds of algorithm training functions we describe. App users in these contexts may be motivated, at least in part, by the promise of tangibly contributing to medical innovation.

SCMD, then, may engage multiple data collection models and purposes. What is important is not a health application's initial function, but the ultimate destination and use of the information it processes. Viewed as a discrete phenomenon, the conceptual value of SCMD derives from two aspects: (a) the pervasive diversity of mobile health data and (b) the highly data-dependent quality of emerging medical innovation modalities, especially algorithm development and training. SCMD draws these features together and, in so doing, also likely presents unique legal and ethical challenges.

### 2. Ethical and legal concerns for the use of SCMD

Collecting and processing health data for any purpose will raise substantial ethical and legal questions. When mobile health data are processed for research or product development, especially when these are not the primary purposes for which the data in question were initially collected, the ethical and legal questions take on a particular resonance. Above, we sketched out a definition of SCMD in which mobile health data are used to train algorithms for medical research or product innovation. This practice would likely raise several significant ethical and legal concerns, notably surrounding the protection of user privacy and autonomy, the application of existing research ethics oversight regimes, and the role of AI law in structuring the use of SCMD. Above, we raised the notion that SCMD could in some settings be used for algorithm training or research purposes without adequate consent. This might be an especially salient concern when smartphone applications are developed and distributed by commercial entities (such as social media firms) or by nonconventional researchers. The rules requiring informed consent for the collection and use of personal data in research might not apply to strictly commercial activities. Nonconventional researchers, moreover, may be unfamiliar with dominant consent regimes and, in consequence, fail to obtain what would otherwise be valid informed consent.

One way that the consent implications of SCMD for algorithms might be realized in legal norms is through privacy protection and data security regulation.^15^ Considering that the SCMD uses we envision require significant data aggregation—often from multiple sources and across several mobile health applications—there is a pronounced risk that even de-identified user information could admit of identification through data combination. This risk is especially pronounced in genetics, where complete anonymization may bei impossible.^16^ SCMD further predicts that personal health data will sometimes change hands, possibly being shared multiple times and between several entities. Each instance of sharing might entail new risks of data breach or potentially identifying data combination. Using SCMD for the kinds of purposes we described above, moreover, might fall into existing regulatory regimes in ways that are uncertain or as yet poorly defined. European data protection laws, for example, set out a short list of acceptable legal bases for the processing of sensitive data, a category that includes health-related data. Perhaps the most relevant legal basis for our purposes, described in Article 9(2) of the *General Data Protection Regulation* (GDPR), is explicit consent.^17^ Additional provisions, notably Articles 9(2)(j) and 89(2) of the GDPR, permit a degree of flexibility when data processing is undertaken for scientific research, and processing app-collected data may be permitted on multiple legal grounds. Recent legislative and regulatory developments in Europe, including proposals for the European Health Data Space, may further impact the legal bases for which mobile health data are collected and used.^18^

Similar rules exist in other jurisdictions, including Canada, where the federal Parliament is presently considering amendments to its national privacy law, the *Personal Information Protection and Electronic Documents Act*.^19^ House of Commons Bill C-27 would broadly align Canada's federal privacy law with the GDPR^20^ and includes several provisions related to the use of personal information in research. In Quebec, there are also provisions pertaining to data subjects’ consent in the recently enacted *Act to modernize legislative provisions as regards the protection of personal information*.^21^ In both the private sector and the public sector, entities or persons may use personal information for a purpose beyond the original consent if the personal information use is necessary for research purposes and if the information is de-identified.^21^ Sharing of personal information can also be nonconsensual if parties sharing information have a written agreement in which a research purpose is detailed.^21^ It is not clear, however, whether using SCMD for algorithm training would constitute a legitimate ground for data processing within the meaning of the GDPR or Bill C-27. It is likewise uncertain what legal basis would serve to justify the *ongoing* processing of personal information, particularly considering that the consent of data subjects to aggregation and repurposing for algorithm training may be ambiguous.

These questions relate to a correlated set of issues: whether existing rules for the conduct of biomedical research or for the regulation of medical devices would apply to SCMD. Traditional regulatory ethics guidance does not directly address novel and technologically dependent modes of data acquisition and sharing.^21^ It is an open question whether using SCMD for algorithm training constitutes “research” within the usual understanding. Even if it does, oversight regimes often apply primarily to conventional health researchers situated in conventional research settings. In Canada and the United States, for example, rules apply formally to researchers working in institutions that receive public funds for that purpose. Though scientists outside research hospitals and university settings are heavily influenced by these regimes—and often abide by their strictures—the rules are not strictly binding.^22^ Commercial entities are likely to be particularly intrigued by SCMD's potential scope of application, while also being potentially excluded from the existing research oversight framework.^23^ It is not obvious that research regulation is conceptually well-suited to these kinds of SCMD use cases, and other ethical and legal instruments might provide a more coherent oversight structure.

Several jurisdictions have begun implementing new legal regimes addressed specifically at controlling the development and use of artificial intelligence.^24^ At one level, these regimes are motivated in significant part by some of the unique risks posed by artificially intelligent data processing. AI is generally understood, for example, to generate acute risks of biased decision-making.^25^ Datasets that are not demographically representative of the populations from which they are derived might work to entrench existing lines of discrimination and inequality.^26^ And though AI laws may try to explicitly address these concerns, the regimes in question may apply inconsistently to the contexts in which SCMD is used, especially if SCMD adopters view their work as primarily constituting research. There is equal uncertainty about the application of medical device regulations to mobile health apps and related technologies: whether mobile apps that process health data ought to be regulated as medical devices remains a highly contested issue. The United States, Canada, and several European jurisdictions have begun regulating medical software under the same regimes applicable to medical devices. While these regimes might generate further complexity for the collection and use of SCMD in algorithm training, it is not obvious that they would apply to smartphone applications or to the collection of medical data solely for the purpose of training a medical algorithm. Significantly more work is required in this space.

### 3. An SCMD research agenda

Largely because practices surrounding SCMD collection and use are not yet well established, there are naturally many unanswered ethical and legal questions in this space. We briefly outlined several of these questions above, but of course there are others. SCMD has great potential to reshape medical research and biomedical innovation but its use also raises a cluster of poorly defined risks. It is essential that scholars anticipate and address these risks at the outset. This could be accomplished in a program of research that documents the degree to which SCMD is being collected and shared to train medical algorithms. This kind of work would be crucial for establishing an evidentiary basis on which further ethical and legal research can be based. One way of achieving this goal would be to create an “app atlas” outlining mobile health applications that collect, process, and share personal medical information for the purposes outlined in this paper. By identifiying mobile health applications available in several jurisdictions, on both the Apple App Store and the Google Play Store, and by reviewing app privacy policies and terms of use to understand how app data are being used, it may be feasible to form an evidentiary basis for further examining the SCMD phenomenon. Though privacy policies and terms of use likely only imperfectly measure how apps use SCMD for algorithm training, we can expect that these documents would nevertheless be a broadly reliable indicator of intended app data uses. Alongside this work, it might be important to understand researcher, app developer, and user perspectives related to SCMD colllection and use. Investigating the attitudes and experiences of these stakeholders would help to further clarify how SCMD is being used and could identify the neede policy mechanisms that would ensure such techniques can be safely adopted. Finally, understanding the privacy law implications of SCMD is essential for crafting policy approaches that would safely and legally structure these activities.

## Conclusion

This paper considers how smartphone-crowdsourced medical data might be useful for training medical algorithms and sets out a brief research agenda for addressing some of the ethical and legal issues associated with this practice. Few recent developments in medical innovation are as potentially disruptive and as understudied as the repurposing of personal health data collected using mobile health applications. Medical AI is likely to significantly reshape medical practice in the coming years and, as it does, will be in search of reliable, representative, and easily accessible training data. Mobile health apps may fruitfully serve as an ample source of such data. SCMD for algorithm training raises an array of legal and ethical questions that, in our view, require scholarly attention to ensure that individual interests are protected and that emerging health information sources can be used in ways that maximally, and safely, promote medical innovation.
